# The RGD region of bone sialoprotein affects metabolic activity in mice

**DOI:** 10.3389/fdmed.2023.1124084

**Published:** 2023-03-10

**Authors:** Karin Nagasaki, Atsuhiro Nagasaki, Jocelyn M. Taylor, Bernice D. Kear, Yinyan Ma, Martha J. Somerman, Oksana Gavrilova

**Affiliations:** ^1^ Laboratory of Oral Connective Tissue Biology, National Institute of Arthritis and Musculoskeletal and Skin Diseases (NIAMS), National Institutes of Health (NIH), Bethesda, MD, United States; ^2^ Department of Periodontology and Endodontology, Tohoku University Graduate School of Dentistry, Sendai, Japan; ^3^ Division of Molecular and Regenerative Prosthodontics, Tohoku University Graduate School of Dentistry, Sendai, Japan; ^4^ Mouse Metabolism Core, National Institute of Diabetes and Digestive and Kidney Diseases (NIDDK), National Institutes of Health (NIH), Bethesda, MD, United States

**Keywords:** mineralized tissues, arginine-glycine-aspartic acid (RGD), bone sialoprotein, metabolic activity, obesity, endocrinology, hyperphagia, extracellular matrix protein

## Abstract

**Introduction:**

Bone sialoprotein (BSP) is a key regulator of mineralized tissue formation. Previously, we generated BSP-KAE knock-in mice (KAEKI mice) by substituting a non-function KAE (lysine-alanine-glutamic acid) for the integrin-binding RGD (arginine-glycine-aspartic acid) sequence and reported a vital role of the BSP-RGD motif in modulating the periodontal ligament (PDL). Specifically, a histological disorganization of the PDL was noted, resulting in a weakened function of the PDL as measured by dynamic mechanical analysis. Intriguingly, also noted was a weight gain as KAEKI mice aged. While several proteins associated with mineralized tissues are reported to affect energy metabolism, the metabolic role of the BSP-RGD region has yet to be elucidated. Here we focus on defining the role of the BSP-RGD region in metabolic activity.

**Methods:**

Body weight, body composition, and caloric intake were measured in wild type (WT) and KAEKI mice. Energy expenditure was estimated using energy balance technique. Epididymal fat, interscapular fat, and liver were harvested for histological analysis. The systemic metabolic phenotype was assessed by sera analyses, insulin tolerance and glucose tolerance tests.

**Results:**

The results showed that KAEKI mice developed mild obesity starting from 13 weeks postnatal (wpn). The increase in body weight correlated with an increase in lean mass and visceral adiposity. Histological examination revealed adipocyte hypertrophy in white epididymal fat and interscapular brown fat in KAEKI vs. WT mice at 17 wpn. Metabolic profiling indicated that KAEKI mice had dyslipidemia and hyperleptinemia but no significant changes in glucose metabolism. Energy balance analyses revealed that hyperphagia preceded weight gain in KAEKI mice.

**Conclusion:**

These data suggest that the RGD region of BSP affects energy metabolism by regulating food intake, with further studies warranted to uncover the underlying mechanisms.

## Introduction

Accumulated data suggest that several proteins associated with mineralized tissues, including proteins containing the arginine-glycine-aspartic acid (RGD) region, affect energy metabolism (1). For instance, osteocalcin, a bone secretory protein, has been reported to act in an endocrine capacity to regulate energy metabolism within adipocytes, hepatocytes, and pancreatic beta cells (2, 3), and osteopontin, a bone-associated RGD containing secretory glycoprotein, has been reported to affect insulin tolerance (4, 5). As a member of the SIBLING (small integrin-binding ligand, N-linked glycoprotein) family, bone sialoprotein (BSP) contains several highly conserved functional motifs, an N-terminal collagen-binding domain, a poly-glutamic acid (poly-E) sequence that nucleates hydroxyapatite, and a C-terminal RGD-integrin binding domain known to promote cell adhesion, migration, and signaling (6). Data from studies using BSP-deficient mice reveal that BSP is a modulator of mineralization (7–13). In brief, BSP-deficient mice have tooth/bone phenotype with alterations in bone homeostasis and mineralization (hypomineralized) and defects in the region of the periodontium, including impairments in the of cementum and surrounding alveolar bone, resulting in a disorganized periodontal ligament (PDL) region, malocclusion and exfoliation of teeth, similar to mice and humans with alkaline phosphatase mutations (14–16). To define the role of the RGD domain of BSP in controlling periodontal tissues, we generated BSP-KAE knock-in (*Ibsp*^KAE/KAE^, hereafter KAEKI) mice by substituting a non-function KAE (lysine-alanine-glutamic acid) sequence for the RGD motif. The results showed an important role of the RGD region of BSP in forming and maintaining the PDL but not in promoting mineralization (6). During our studies with these mice, we noted that the KAEKI mice gained more weight than controls as they aged. This was an unexpected finding as the BSP-deficient mice had lower body weight and size than their wild-type (WT) counterparts with no difference in percentage of fat mass between the two genotypes (8). Thus, it appeared that, beyond bones and teeth, the BSP-RGD region may play a role in systemic metabolic activity.

This observation led us to initiate studies to further elucidate the role of the RGD region of BSP in modulating metabolic activity. Importantly, obesity is a major health problem worldwide as it is associated with a number of chronic diseases, including type 2 diabetes, dyslipidemia, cardiovascular diseases and other disorders (17). The fundamental cause of obesity is a long-term energy imbalance between caloric intake and energy expenditure. Alterations of glucose and lipid metabolism have also been reported to influence bone homeostasis (18). Skeletal tissue growth and remodeling are energy-consuming processes tightly coupled with the regulation of systemic energy metabolism and reproduction (19). Numerous hormones, such as estrogen, testosterone, parathyroid hormone, insulin, adipokines (e.g., leptin, resistin, adiponectin, tumor necrosis factor-α), vitamin D, and neuropeptides modulate bone metabolic activity (18, 20–26). Further, bone marrow adipose tissue, located in close proximity to skeletal lineage cells, has been shown to affect bone metabolism (27–30). Specifically, expansion of this depot, observed with aging, obesity, diabetes, and anorexia nervosa, is often inversely associated with bone mineral density. Bone marrow adipocytes and osteoblasts share a common precursor, mesenchymal stem cells, and thus an imbalance between adipogenesis and osteogenesis, such as a consequence of pathological conditions, may contribute to bone loss.

Added to the growing evidence that specific factors control cell fate toward an adipocyte vs. osteoblast pathway, there exists credible evidence that proteins produced by mineralized tissues, including several RGD-containing proteins, affect the activity of tissues at distant sites (1, 20, 31–34). However, the specific role of these proteins at distant sites is not fully understood. Therefore, in this study we utilized a KAEKI mouse model to examine the role of the BSP-RGD region in systemic metabolic activity.

## Materials and methods

### Mice

Animal studies were approved by the NIAMS and NIDDK Animal Care and Use Committees (NIH, Bethesda, MD). KAEKI mice (previously reported as *Ibsp*^KAE/KAE^ mice) were generated by CRISPR/Cas9 as reported previously (6). WT and KAEKI mice were maintained on a C57BL/6 background as described previously (6). Mice were housed at ∼22°C with a 12–12 h light-dark cycle and fed soft gel (DietGel® 31M, 1.91 kcal/g, ClearH_2_O, Inc, Westbrook, ME) and normal chow (NIH-07, 3.1 kcal/g, Envigo Inc, Madison, WI) diet to ensure that malocclusion, attributable to the impaired periodontal complex reported in global BSP knockout mice, which did not occur and thereby affect food intake (35). For consistency, male mice were used for all experiments. A cross-sectional study was performed to measure body weight from 1 to 17 weeks postnatal (wpn), using group housed mice, which were randomly selected throughout the 17 weeks (*n* = 4 per genotype for each time point). A separate cohort of group-housed mice was used for measuring body weight, body length, fat pad and liver weights at 17 wpn, as well as for histological analysis of epidydimal and brown fat. Another cohort of singly housed mice was used for measuring energy balance, insulin tolerance and glucose tolerance.

### Measurement of body composition, body length, food intake and energy expenditure

WT (*n* = 5) and KAEKI (*n* = 6) mice were singly housed from 6 to 15 weeks postnatal (wpn). Body weight, body composition, and caloric intake were measured once a week. Total metabolizable caloric intake was calculated from the combined intake of chow and soft gel diets. Body composition (fat mass and fat-free mass) was measured by time domain EchoMRI 3-in-1 (Echo Medical Systems, Houston, TX). Energy expenditure was estimated from the metabolizable caloric intake, corrected for the change in caloric content of the mouse (from the change in body composition over the measurement interval using caloric equivalents of fat mass 9.4 kcal/g and fat-free mass 1.0 kcal/g) (36). Body length (nose-to-anus distance, mm) was assessed immediately after euthanasia at 17 wpn. Body mass index (BMI) was calculated as body weight (kg)/body length^2^ (m^2^).

### Insulin and glucose tolerance tests

An insulin tolerance test (ITT) was performed at 16 wpn by injecting non-fasted mice with insulin (Humulin R, Eli Lilly, Indianapolis, IN, 0.75 u/kg, i.p.). Tail blood glucose concentrations were measured at 0, 15, 30, 45, and 60 min using glucose meter Contour (Ascensia, Parsippany, NJ). A glucose tolerance test (GTT) was conducted at 17 wpn by injecting mice with 20% glucose (2 g/kg, i.p.), following an overnight (16 h) fast, with blood glucose measured at 0, 15, 30, 60, and 120 min. ITT and GTT tests were performed on the cohort of mice used for body composition and energy balance analyses. HOMA-IR (Homeostasis Model Assessment of Insulin Resistance) index was calculated as described previously (37).

### Tissue/blood collection and analyses

Another cohort of mice was prepared for blood and tissue analyses. Non-fasted mice were euthanized at 17 wpn by cervical dislocation, followed by collection of blood directly from the heart. Epididymal fat pad (white fat; WT *n* = 6, KAEKI *n* = 6), interscapular brown fat (WT *n* = 5, KAEKI *n* = 5), and liver (WT *n* = 6, KAEKI *n* = 6) were harvested after blood collection. Percent (%) epididymal fat pad and % liver were calculated by dividing tissue weight by body weight per mouse (WT *n* = 6, KAEKI *n* = 6).

Plasma chemistry tests: glucose (WT *n* = 11 KAEKI *n* = 12), triglycerides (WT *n* = 10, KAEKI *n* = 12), and cholesterol (WT *n* = 10, KAEKI *n* = 12) were performed by the Department of Laboratory Medicine, NIH Clinical Center. Insulin (WT *n* = 8, KAEKI *n* = 10) and leptin (WT *n* = 7, KAEKI *n* = 8) levels in sera were measured by enzyme-linked immunosorbent assay according to the manufacture's protocol (Mouse Leptin ELISA Kit, RAB0334, Sigma-Aldrich; Ultra-sensitive Mouse Insulin ELISA, #90080, Crystal Chem).

### Histology

Tissues were fixed in 10% neutral buffered formalin for 24 h and paraffin embedded for serial 5*μ*m sections. Hematoxylin and eosin (H&E) staining was conducted with Harris' hematoxylin and eosin Y 1% alcoholic solution (Thermo Fisher Scientific, Waltham, MA) as described previously (38). For measurement of white adipocyte size, 20 × magnification H&E staining images of epididymal fat pat were measured manually using Rebel Hybrid Microscope (ECHO, San Diego, CA), and cell size was expressed in mm^2^ (*n* = 4). For measurement of intrascapular brown adipocyte size, 60 × magnification H&E staining images were analyzed using ImageJ (NIH), and data were expressed as percent of area filled with lipid (*n* = 5).

### Statistical analysis

The results are expressed as mean ± standard deviation. Data were analyzed using *t*-test (Prism v.7.04, GraphPad Software, La Jolla, CA). For all tests, *α* = 0.05.

## Results

### Mice lacking the BSP-RGD region exhibit obesity with age

**Figure 1:**
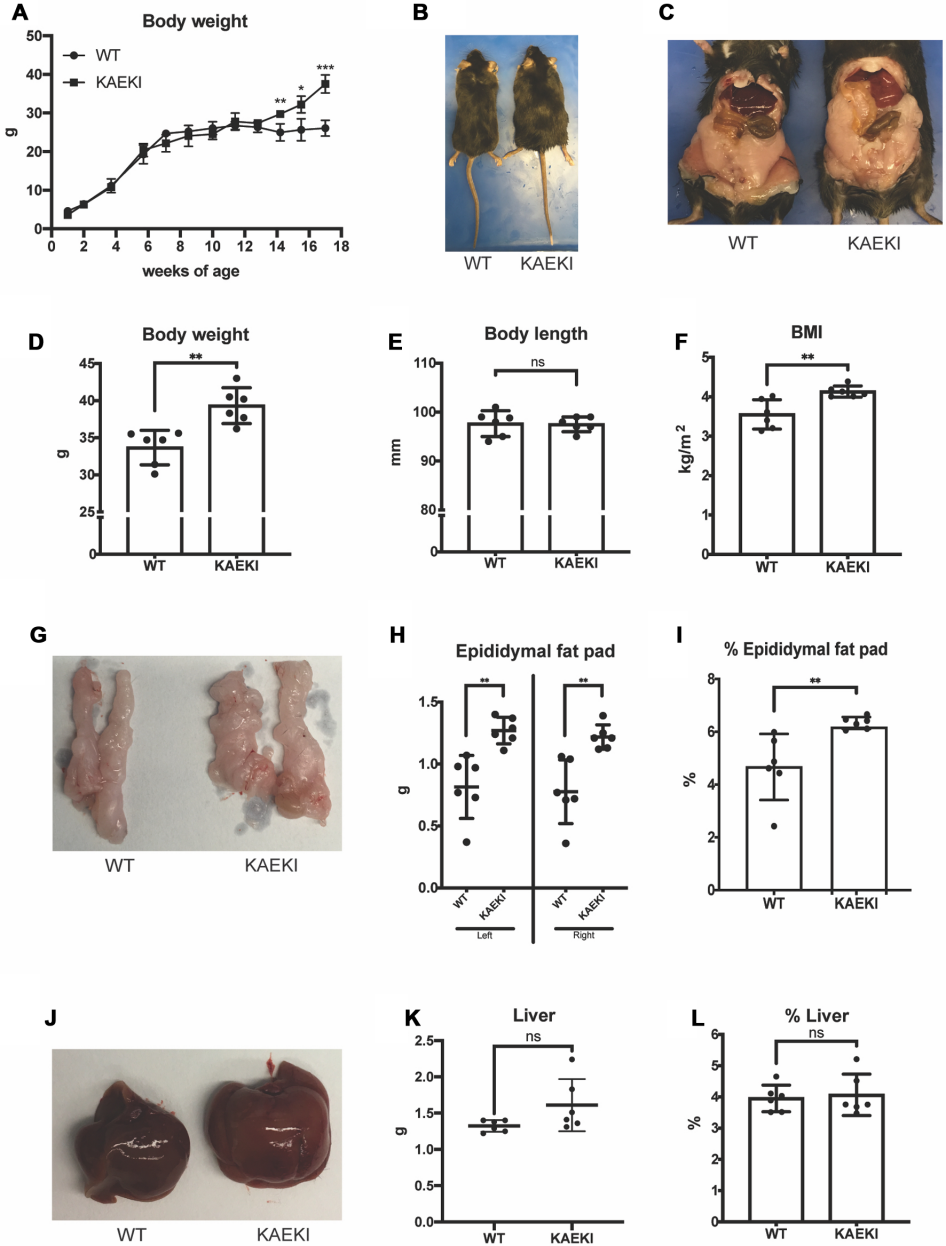
Mice lacking the BSP-RGD region develop obesity as they grow. (**A**) Body weights of WT (*n* = 4) and KAEKI (*n* = 4) mice from 1 to 17 wpn. Data were collected from a cross-sectioned study (*n* = 4/genotype/time point). (**B**) Dorsal view of representative WT and KAEKI mice at 17 wpn. (**C**) Ventral view of representative WT and KAEKI mice at 17 wpn after removal of abdominal walls. (**D–E**). Body weight and body length measured in a different set of mice (*n* = 6/group). (**F)** BMI at 17wpn (*n* = 6/group). (**G–I**) Appearance, weight, and % weight of epidydimal fat pads at 17 wpn (*n* = 6/group). (**J–L**) Appearance, weight, and % weight of liver at 17 wpn (*n* = 6/group). Results are expressed as mean ± standard deviation. *P < 0.05; **P < 0.01; ***P < 0.001; ns, not significant by *t*-test. Male mice were used for all experiments.

To examine the role of the BSP-RGD in systemic metabolic activity, we first followed body weight changes in KAEKI and WT mice during development (Figure 1A). Growth rates were similar in both genotypes up to 13 wpn. However, beyond that point, KAEKI mice gained weight more rapidly and by 17 wpn were approximately 17% heavier than controls with no difference in body lengths between genotypes (Figures 1B, D). Consistent with significantly increased body weight, KAEKI mice developed more visceral fat (Figure 1C), had higher BMI (Figure 1F), and displayed significantly larger (56%) epididymal fat pats (Figures 1G–I) than WT mice. Liver weight was not significantly different between genotypes (Figures 1J–L). Taken together, these data indicate that KAEKI mice develop mild obesity with age. We also noted a difference in body weight of WT mice depending on the cohort used (see Figure 1A vs. Figure 1D); however, this did not alter the significant difference in body weight between WT vs. KAEKI mice.

### KAEKI mice display adipocyte hypertrophy

**Figure 2:**
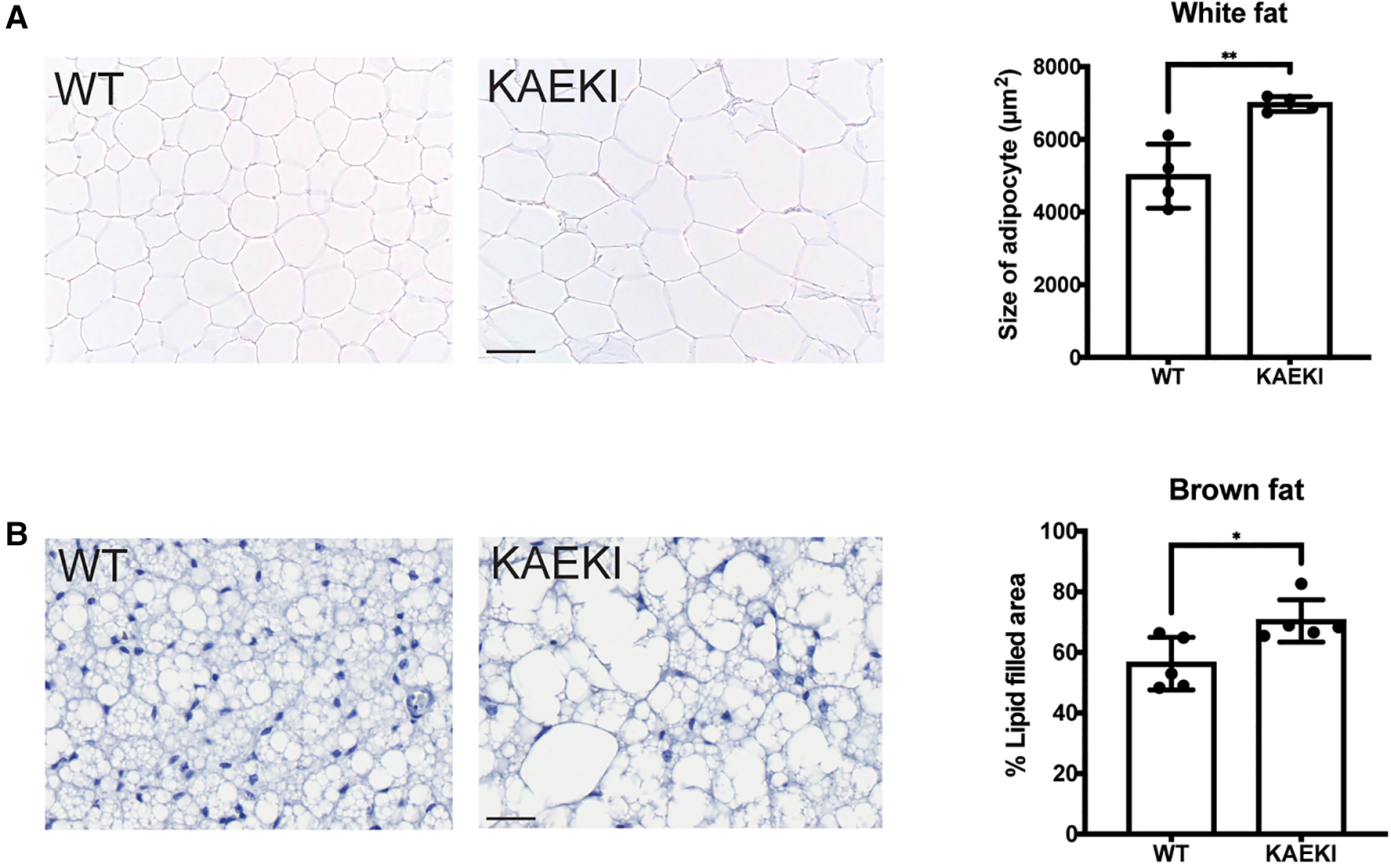
KAEKI mice display adipocyte hypertrophy. Histological appearance (H&E staining) of white (epidydimal) fat (**A**) and interscapular brown fat (**B**) in WT and KAEKI mice at 17 wpn. For brown fat, relative cell size is expressed as % area filled with lipid. Results are expressed as mean ± standard deviation. *P < 0.05; **P < 0.01 by *t*-test. Scale bar:100 µm for white fat and 20 µm for brown fat. Male mice were used for all experiments.

Expansion of adipose tissue occurs through an increase in adipocyte cell size (hypertrophy) and/or cell number (hyperplasia), with hypertrophic expansion of white fat associated with more severe metabolic dysfunction (39). Therefore, we next examined the histological appearance of white fat (energy storage tissue) and brown fat (thermogenic tissue). The size of both white and brown adipocytes was increased in KAEKI mice compared to WT mice (Figures 2A, B). There were also notable differences in the appearance of brown adipocytes. Typically, wild-type brown adipocytes contained multiple small lipid droplets. In contrast, the KAEKI brown fat adipocytes appeared heterogeneous in size, containing predominantly large fat droplets, a phenotype often observed in obese mice with reduced cold-induced thermogenesis (40) (Figure 2B).

### KAEKI mice display dyslipidemia and hyperleptinemia

**Figure 3:**
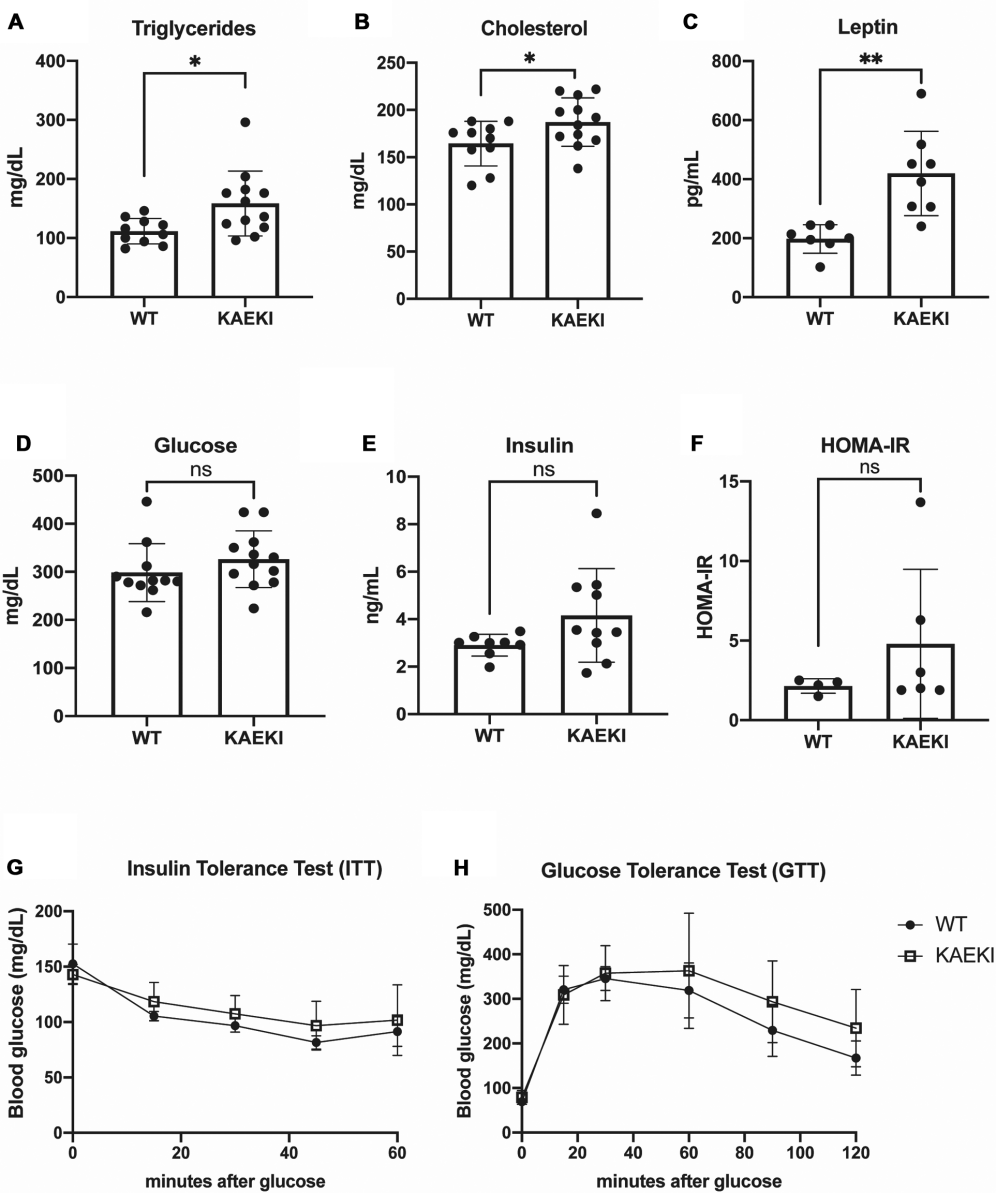
KAEKI mice display dyslipidemia and hyperlipidemia. (**A–E**) Blood samples were obtained by cardiac puncture at 17 wpn from WT and KAEKI mice. (**A**) Total triglyceride: WT (*n* = 10) and KAEKI (*n* = 12). (**B**) Total cholesterol: WT (*n* = 10) and KAEKI (*n* = 12). (**C**) Leptin:WT (*n* = 7) and KAEKI (*n* = 8). **D**. Glucose:WT (*n* = 11) and KAEKI (*n* = 12). **E**. Insulin: WT (*n* = 8) and KAEKI (*n* = 10). **F**. HOMA-IR performed at 17 wpn: WT (*n* = 4) and KAEKI (*n* = 6). (**G**) Insulin tolerance test (ITT) performed at 16 wpn: WT (*n* = 5) and KAEKI (*n* = 6). **H**. Glucose tolerance test (GTT) performed at 17 wpn: WT (*n* = 5) and KAEKI (*n* = 6). Results are expressed as mean ± standard deviation. *P < 0.05; **P < 0.01; ns = not significant by *t*-test. Male mice were used for all experiments.

The observed obesity, along with hypertrophy of white adipocytes in KAEKI mice, led us to hypothesize that lack of the BSP-RGD region affects systemic metabolism. Sera analyses revealed that circulating levels of triglycerides and cholesterol were significantly increased in KAEKI mice, indicating a dysregulation of lipid metabolism (Figures 3A, B). Levels of leptin, an adipokine produced in proportion to fat mass and a regulator of energy balance by inhibiting food intake, were also significantly increased (Figure 3C). However, non-fasted serum glucose and insulin levels, HOMA-IR (insulin resistance index), insulin tolerance and glucose tolerance were not significantly different between genotypes (Figures 3D–H). Thus, mice lacking BSP-RGD signaling display dyslipidemia and hyperleptinemia with no significant changes in systemic glucose metabolism.

### KAEKI mice display hyperphagia

**Figure 4:**
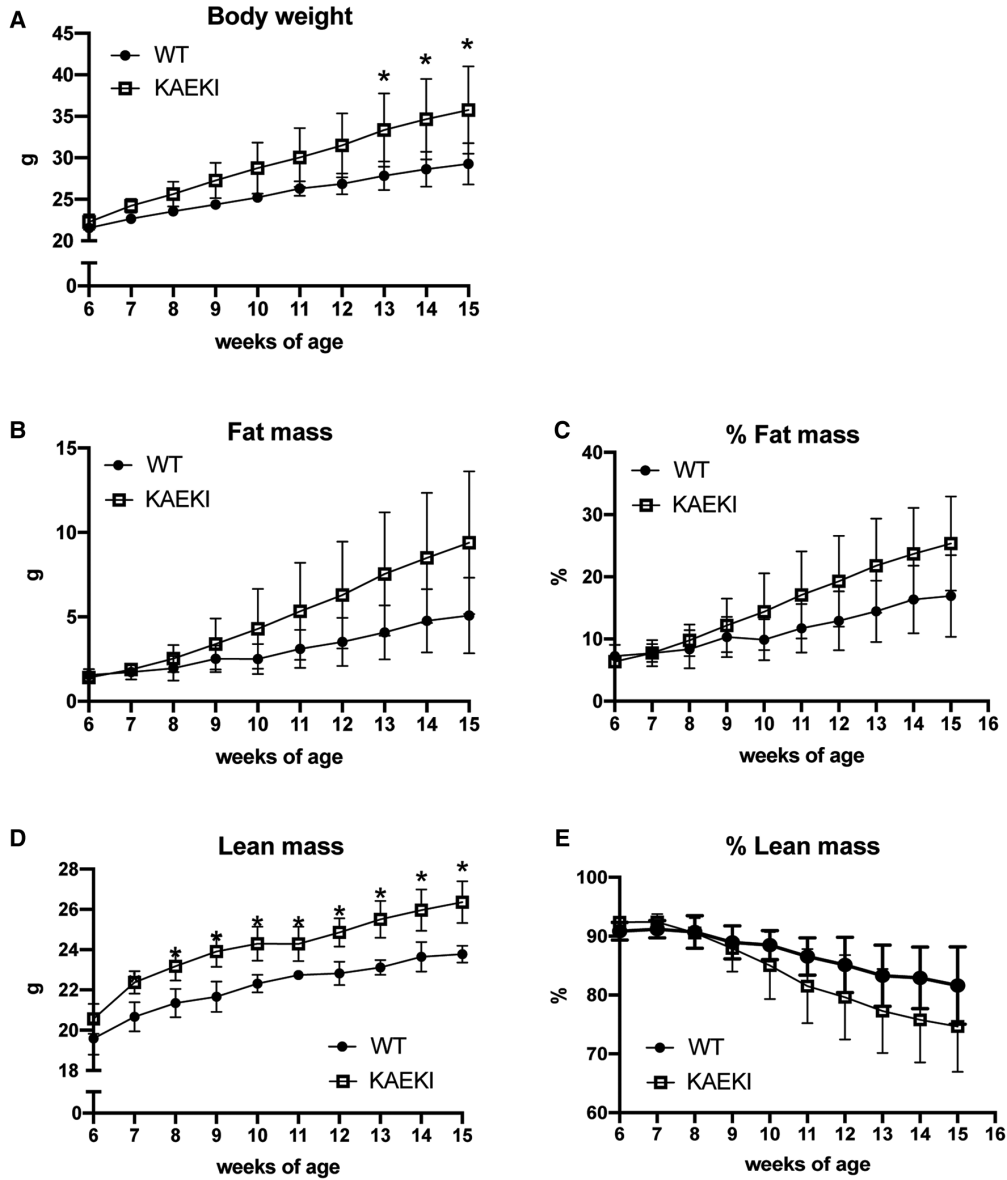
Lack of the BSP-RGD region alters body composition. Body weight (**A**), fat mass (**B**), % fat mass (**C**), lean mass (**D**), and % lean mass (**E**) were measured weekly in a cohort of WT (*n* = 5) and KAEKI (*n* = 6) mice. Results are expressed as mean ± standard deviation. *P < 0.05 by *t*-test. Male mice were used for all experiments.

Obesity results from an imbalance between energy intake and energy expenditure. To gain further insight into the cause of obesity in KAEKI mice, we analyzed energy balance in a separate cohort of mice by monitoring caloric intake and changes in body composition over a period of 9 weeks. Consistent with previous findings, KAEKI mice showed significantly increased body weight (Figure 4A); however, the noted increase in fat mass did not reach significance due to a large variation in individual mice (*p* = 0.15, Figures 4B, C). KAEKI mice also had significantly higher lean mass volume vs. WT mice, although % lean mass, a proportion of lean mass to total body weight, in KAEKI mice tended to be lower than controls (Figures 4C, D), suggesting that both fat and lean mass contribute to the increased weight gain in mice lacking BSP-RGD signaling.

**Figure 5:**
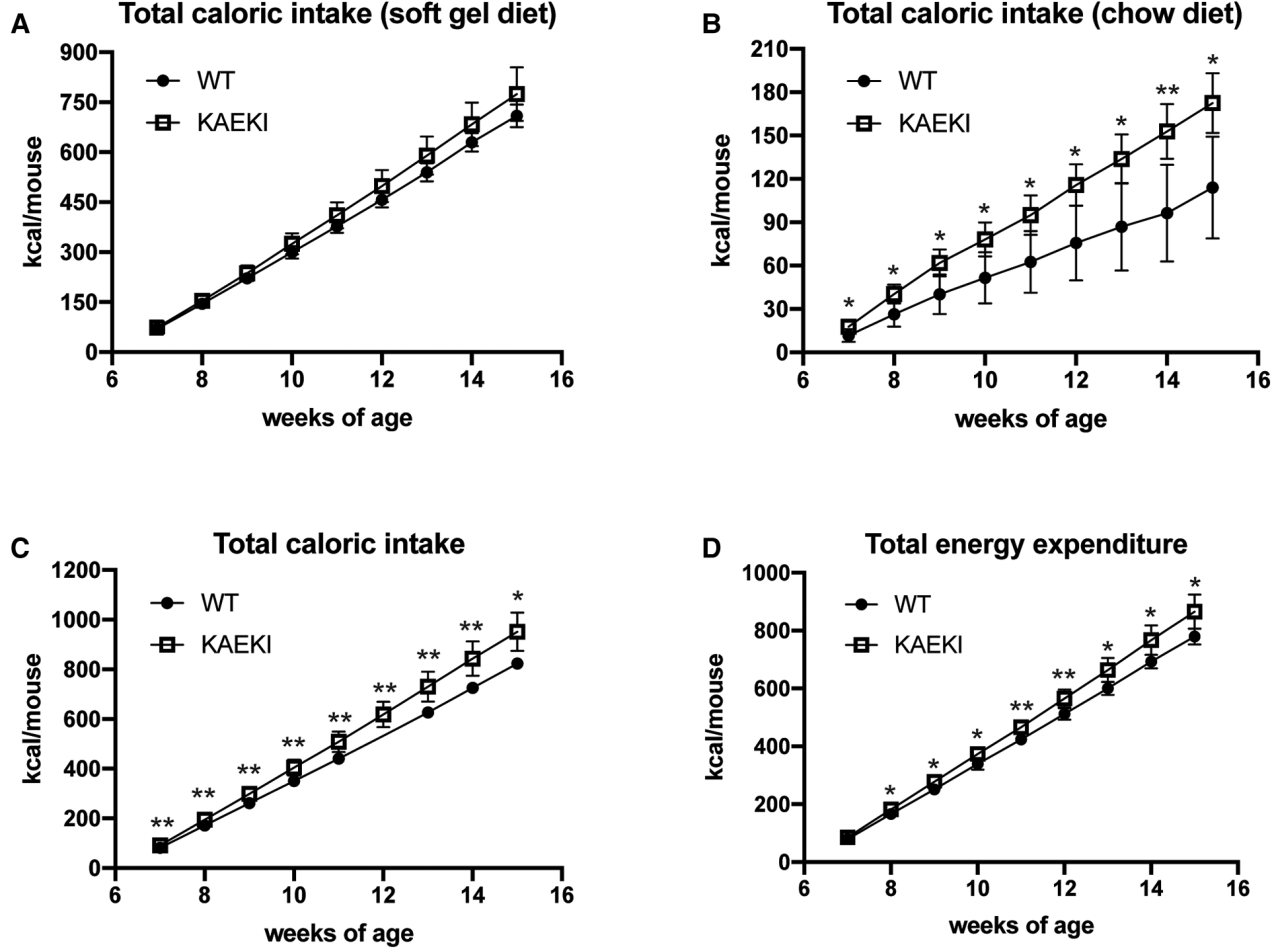
KAEKI mice exhibit hyperphagia. Intake of soft gel (**A**) and chow (**B**) diets was measured weekly in the cohort of WT (*n* = 5) and KAEKI (*n* = 6) mice used for body composition analysis (see Figure 4). Data are expressed as cumulative caloric intake. (**C**) Total caloric intake is a combined intake of soft gel and chow diets. (**D**) Total energy expenditure estimated from total caloric intake, corrected for the change in caloric content of the mouse. Results are expressed as mean ± standard deviation. *P < 0.05; **P < 0.01; ns = not significant by *t*-test. Male mice were used for all experiments.

To calculate total caloric intake, we measured cumulative consumption of soft gel diet and chow diet. Both KAEKI and WT mice obtained most of their calories from the soft diet (81% and 86%, respectively, at 15 wpn) with no significant difference between genotypes (Figure 5A). In contrast, the intake of chow was 58% higher in KAEKI mice compared to controls by 15 wpn (Figure 5B), leading to 16% higher total caloric intake by 15 wpn (Figure 5C). This difference was noted early on, 7 wpn, where KAEKI mice had a 12% higher total caloric intake vs. controls. Further, the estimated total energy expenditure was slightly higher in the KAEKI mice than WT mice, consistent with their increased lean mass (Figure 5D). Taken together, these data suggest that hyperphagia, but not hypometabolism, was the primary cause of obesity in KAEKI mice.

## Discussion

An intriguing finding of accelerated weight gain in aging KAEKI mice spurred us to examine the role of the BSP-RGD region in metabolic activity. When we first made this observation, we noted increased body weight in both male and female mice; however, we decided to focus on male mice at this time. Here we show that male mice lacking BSP-RGD signaling develop mild, adult-onset obesity associated with hyperphagia, increased lean mass and visceral adiposity, and adipocyte hypertrophy. Although obesity is commonly associated with metabolic syndrome, including hyperglycemia, insulin resistance and abnormal circulating cholesterol or triglyceride levels, KAEKI mice displayed only a small elevation of serum cholesterol and triglyceride levels and no significant changes in glucose metabolism, consistent with the relatively mild obesity observed in KAEKI mice.

The body weight phenotype of KAEKI mice is strikingly different from the phenotype of BSP null mice, which exhibit lower body weight and size than their WT littermates under standard chow conditions, with no difference in percentage of fat mass between the genotypes (8). Feeding BSP null mice a soft diet improved malocclusion, attributed to a severe periodontal phenotype, and normalized their body weight and long bone length, suggesting that malocclusion might be the primary reason for the reduced weight gain in the BSP-deficient mice maintained on a hard diet (35). Therefore, since we did not have any prior knowledge of the role of the BSP-RGD in maintaining normal occlusion, all mice in this study were given *ad libitum* access to a soft diet and a regular rodent chow, although we recognize that the texture of the diet may affect preference for chow vs. soft gel diet. As we reported previously (6), compared to the BSP null mice (10), the KAEKI mice had a mild periodontal phenotype including a disorganized and dysfunctional PDL and increased osteoclasts along the alveolar bone surface, without disruption of the tooth and bone formation. Even the mild PDL phenotype exhibited in KAEKI mice may affect the response to occlusal loads during mastication (41); however, we do not believe this is the case since KAEKI mice consumed equal amounts of the soft diet and more chow diet vs. WT mice without developing malocclusion. For future studies, standard chow diet will be considered as the diet for both genotypes.

Obesity results from a long-term imbalance between energy intake and energy expenditure, which may be caused by a combination of overconsumption of highly caloric and palatable foods, low physical activity and reduced basal metabolism (17). Studies in mice and humans showed that obesity and aging are also associated with reductions in the amount and activity of thermogenic adipose tissue (42). The histological appearance of brown fat in KAEKI mice, including adipocyte hypertrophy, is consistent with the appearance of dormant brown fat observed in obese mice deficient in cold-induced thermogenesis (40). In mice housed at room temperature (22°C), cold-induced thermogenesis (mainly mediated by brown fat) accounts for about 30% of total energy expenditure (40). Thus, impaired brown fat thermogenesis can potentially reduce total energy expenditure and lead to weight gain in a mouse. However, the estimated total energy expenditure was slightly higher, not lower, in KAEKI mice than in WT mice, consistent with their increased lean mass. In contrast, KAEKI mice showed significantly increased caloric intake as early as 7 wpn, weeks before detectable changes in body weight gain. Interestingly, the increase in total caloric intake observed in KAEKI mice was mainly driven by higher consumption of a chow diet, which has ∼60% higher caloric density than a soft gel diet. In designing future studies, it would be of value to explore how KAEKI mice respond to high-fat or high-sugar diets. Taken together, our data suggest that hyperphagia, but not hypometabolism, is the primary cause of obesity in mice lacking BSP-RGD signaling.

One outlier in concluding a KAEKI hyperphagia phenotype is our sera analyses, which revealed that KAEKI mice had significantly increased leptin levels. Leptin, a hormone produced by adipocytes in proportion to fat mass, regulates energy balance primarily by inhibiting food intake (43, 44). Elevated serum leptin in the presence of obesity could be a marker of reduced leptin sensitivity (45) and may contribute to the obesity phenotype of KAEKI mice. However, since these mice displayed hyperphagia prior to detectable changes in fat mass, it is unlikely that hyperleptinemia is a primary cause of obesity in this model.

Skeletal tissue growth and remodeling are energy-consuming processes tightly coupled with regulation of energy metabolism and reproduction (19). Osteopontin, another bone-secreted RGD-containing protein, is considered to be associated with obesity, insulin resistance and type 2 diabetes with a reported vital role in modulating inflammation within many tissues including adipose tissue (4, 5, 46). Moreover, osteocalcin, a protein specifically expressed by osteoblasts, has been reported to regulate energy metabolism via effects on adipocytes, hepatocytes, and pancreatic beta cells (2, 3). While further studies are needed to better understand the influence of the RGD region of BSP on metabolic activity, the results reported here add to existing evidence that proteins within mineralized tissues influence metabolic activity at other sites (1). The metabolic homeostasis of higher organisms relies on precise sensing of the energy state of the body and a coordinated response of multiple organs to nutritional demands and environmental changes. The central nervous system plays an important role in regulating all aspects of metabolism, including energy intake, utilization, and storage (47–49). One way peripheral tissues communicate with the brain is via secreted factors, including proteins, hormones, cytokines, and metabolites (26, 39, 50–53). BSP is a multifunctional extracellular matrix protein abundant in bone, cementum, and dentin (54–56). Although low levels of *Ibsp* mRNA have been detected in the mouse brain (https://www.ncbi.nlm.nih.gov/gene/3381, http://www.informatics.jax.org, https://www.ebi.ac.uk), there is no evidence of BSP expression in the areas of the brain regulating energy metabolism. A small amount of BSP is found in the circulation (57) but it is likely to be just a marker of bone turnover as BSP is not known to have an endocrine function. Thus, the metabolic phenotype of KAEKI mice is more likely caused by impaired BSP-RGD signaling within mineralized tissues rather than its direct effect at distal sites. For example, affecting the levels of proteins within mineralized tissues is reported to affect energy metabolism (1, 20, 31, 32).

In this regard, as mentioned above, existing data provide credible evidence that proteins produced by mineralized tissues, including several RGD-containing proteins, affect the metabolic activity of tissues at distant sites, including regulation of body weight, energy expenditure, insulin secretion and insulin sensitivity (1, 20, 31–33). To date, the osteoblast-derived lipocalin-2 is the only known bone-derived factor that has been shown to regulate food intake directly by activating melanocortin 4 receptor-dependent anorexigenic pathway in the hypothalamus (58). Further studies are needed to determine if the expression of lipocalin 2 and other known bone-derived factors is altered in mice lacking BSP-RGD signaling.

Taken together, our data suggest that, beyond its role in bones and teeth, the RGD region of BSP contributes to systemic metabolic activity by controlling food intake, consequently increasing caloric storage within adipocytes, resulting in white and brown fat hypertrophy and overall gain of fat and lean mass. Further studies are warranted to determine the mechanisms by which the RGD region of BSP, as well as other RGD-containing proteins, modulate systemic metabolic activity.

## Data Availability

The original contributions presented in the study are included in the article, further inquiries can be directed to the corresponding author.
